# Draft Genome Sequence of Listeria monocytogenes Serovar 1/2a Strain IZSAM_Lm_14-16064, Isolated from an Italian Cooked Ham in 2014

**DOI:** 10.1128/MRA.00558-20

**Published:** 2020-07-02

**Authors:** Valeria Di Lollo, Massimiliano Orsini, Vicdalia Acciari, Cesare Cammà, Patrizia Centorame, Gabriella Centorotola, Valentina Curini, Adriano Di Pasquale, Antonio Rinaldi, Stefano Pongolini, Marina Torresi, Francesco Pomilio

**Affiliations:** a National Reference Laboratory for *Listeria monocytogenes*, Istituto Zooprofilattico Sperimentale dell’Abruzzo e del Molise G. Caporale, Teramo, Italy; b National Reference Centre for Whole Genome Sequencing of microbial pathogens: database and bioinformatic analysis, Istituto Zooprofilattico Sperimentale dell’Abruzzo e del Molise G. Caporale, Teramo, Italy; c Istituto Zooprofilattico Sperimentale delle Venezie, Legnaro, Italy; d Istituto Zooprofilattico Sperimentale della Lombardia e dell’Emilia Romagna, Parma, Italy; Georgia Institute of Technology

## Abstract

In this report, the draft genome sequence of Listeria monocytogenes serovar 1/2a strain IZSAM_Lm_14-16064, isolated in Italy from a cooked ham, is announced. The genome is similar to that of a clinical strain isolated in 2014.

## ANNOUNCEMENT

Listeria monocytogenes is a ubiquitous Gram-positive bacterium representing the causative agent of listeriosis, a disease that afflicts both humans and animals. This organism continues to be one of the most important foodborne psychrotrophic pathogens due to its ability to survive under several environmental conditions, such as low pH, refrigeration temperature, and high NaCl concentrations (1).

As members of the National Reference Laboratory for Listeria monocytogenes, we conduct retrospective studies and sequence comparisons within the national L. monocytogenes genome sequence database in order to detect clusters and persistent strains and to link genetic traits to epidemiological evidence.

Here, we report the characterization of an L. monocytogenes serogroup 1/2a isolate (strain IZSAM_Lm_14-16064) detected in a cooked ham collected in northern Italy in 2014.

The strain was isolated from cooked ham according to ISO 11290-1 (2). One colony, after an overnight incubation at 37°C on a blood agar plate, was picked and dissolved in 300 μl of nuclease-free water. Then, 100 μl of 20 mg/ml lysozyme was added and incubated for 2 h at 56°C. Finally, 300 μl of the suspension was transferred to the cartridges provided by the Maxwell 16 cell DNA purification kit (Promega Italia Srl, Milan, Italy) according to the manufacturer’s protocol. After species confirmation by PCR assay (3), 1 ng of genomic DNA was used for library preparation using the Nextera XT DNA library prep kit (Illumina, San Diego, CA) according to the manufacturer’s protocol. Deep sequencing was performed on the NextSeq 500 platform (Illumina) with the NextSeq 500/550 midoutput reagent cartridge v2 (300 cycles, standard 150-bp paired-end reads).

The sequencing returned 3,996,151 read pairs (2 × 150 bp with an average length of 130 bp), corresponding to a theoretical coverage of about 370×. Quality control, trimming, and preliminary genome assembly were carried out on the Orione platform (4) using “Fastq quality and positional trimming” and SPAdes v3.5 (5) with default parameters for 2 × 150-bp sequencing chemistry. Default parameters were used for all software unless otherwise specified.

The preliminary assembly of the entire genome showed 99.38% sequence identity, calculated by the OrthoANI v1.2 tool (6), to an L. monocytogenes 1/2a isolate (GenBank accession no. CP013919) identified by our group during an Italian foodborne outbreak investigation in 2008 (7) and 99.93% to 99.95% sequence identity, calculated by the OrthoANI v1.2 tool, to a group of isolates detected in Canada (i.e., CP007019, CP018685, CP001604, CP007008, CP007017, CP006861, CP007007, CP007011, CP009258, and CP008836) from clinical patients and food samples collected from 1998 to 2010. The closest reference genome was chosen by submitting raw contigs to the KmerFinder Web server (https://cge.cbs.dtu.dk/services/KmerFinder/). It returned the L. monocytogenes R479a (NZ_HG813247) genome as the closest one. As a guide for scaffolding, Abacas software v1.3 (8) was used. Gaps in the returned pseudomolecule were filled by running several rounds of GapFiller v2.1.1 (9). Finally, the draft sequence was refined by using Pilon v1.23 (10) and evaluated by QUAST v5.0.2 (11).

The final assembly, consisting of 4 contigs (average length, 745,950 bp; *N*_50_, 1,628,990 bp), was submitted to GenBank and annotated by the NCBI staff using the Prokaryotic Genome Annotation Pipeline (PGAP; https://www.ncbi.nlm.nih.gov/genomes/static/Pipeline.html). The annotation returned 2,983 genes, 2,877 coding sequences, 5 full rRNA operons, 19 pseudogenes, 8 frame-shifted genes, 1 CRISPR array, 1 noncoding RNA (ncRNA), and 71 tRNAs. The OrthoANI tool (6) gave evidence of a global sequence identity of 99.46% against the epidemiologically linked clinical strain (CP013919).

### Data availability.

The complete genome sequence of this isolate has been deposited in the NCBI assembly database with the accession no. GCF_001280245.1. Raw reads were uploaded to the SRA database under the accession no. SRR2182156.
